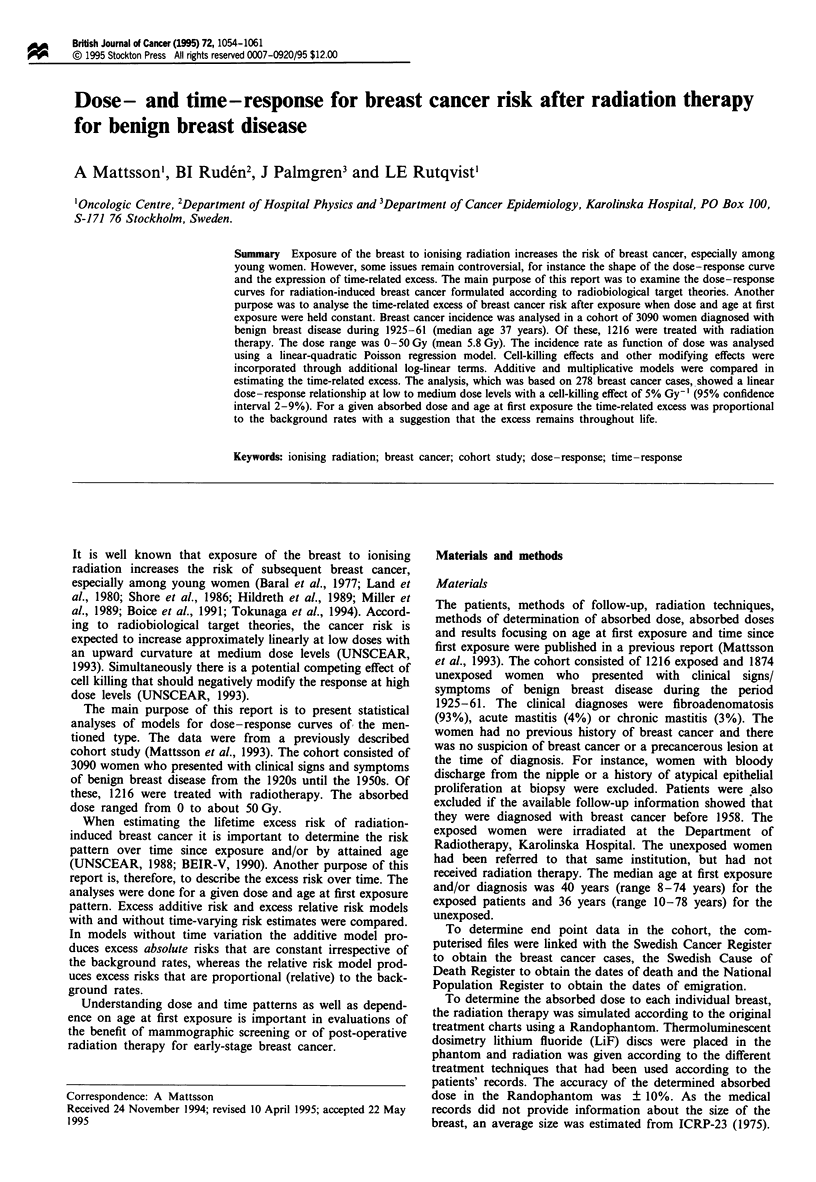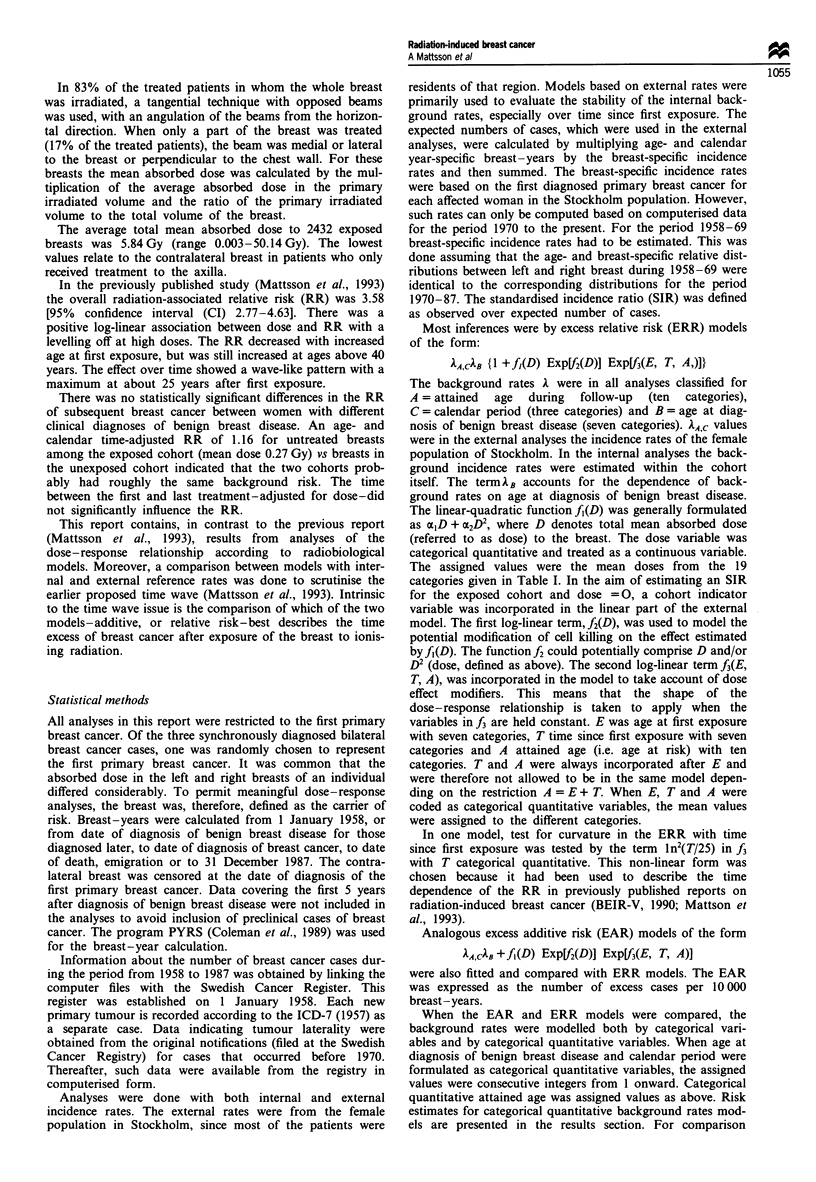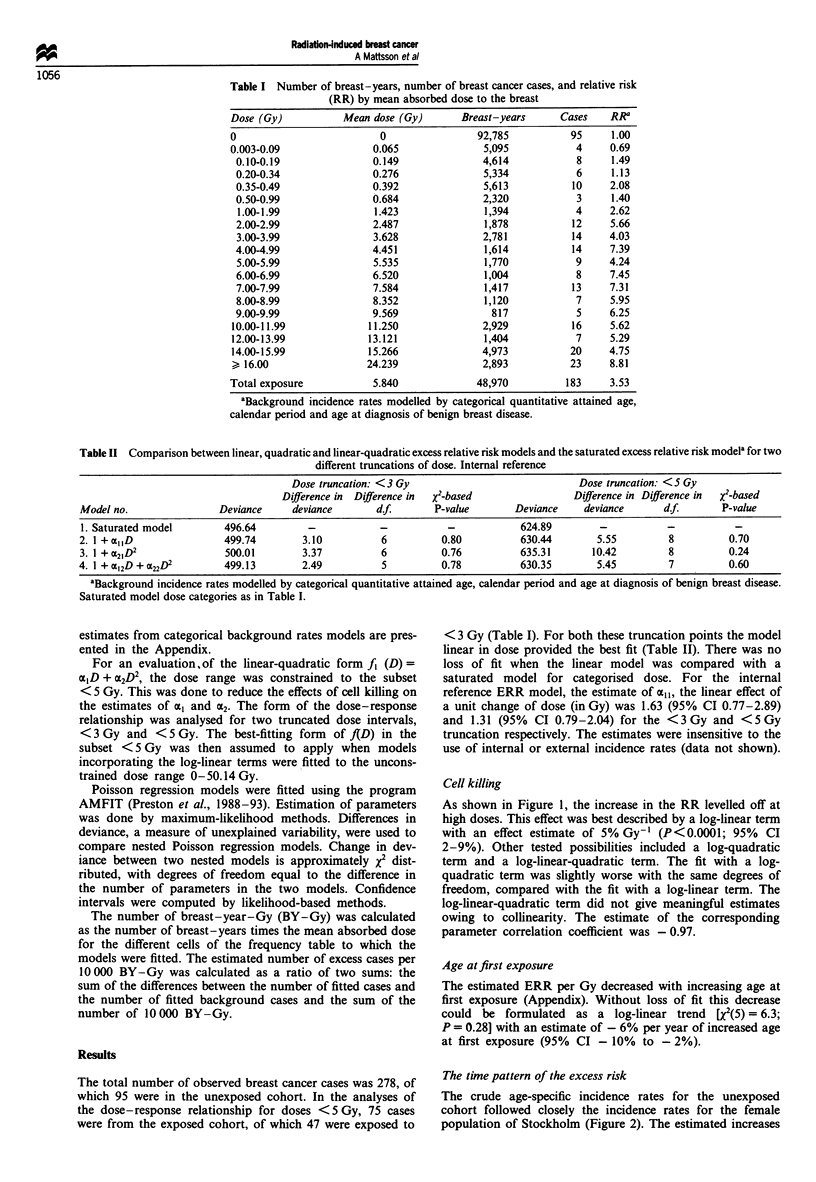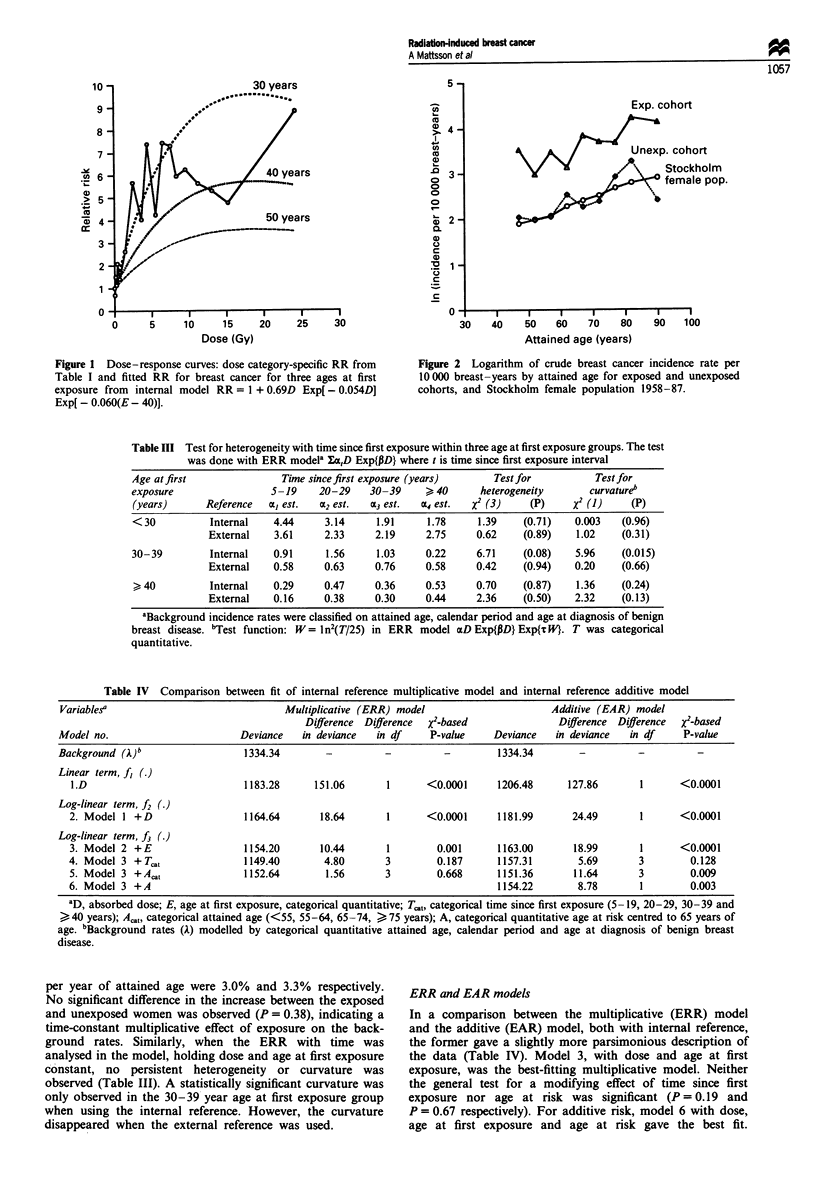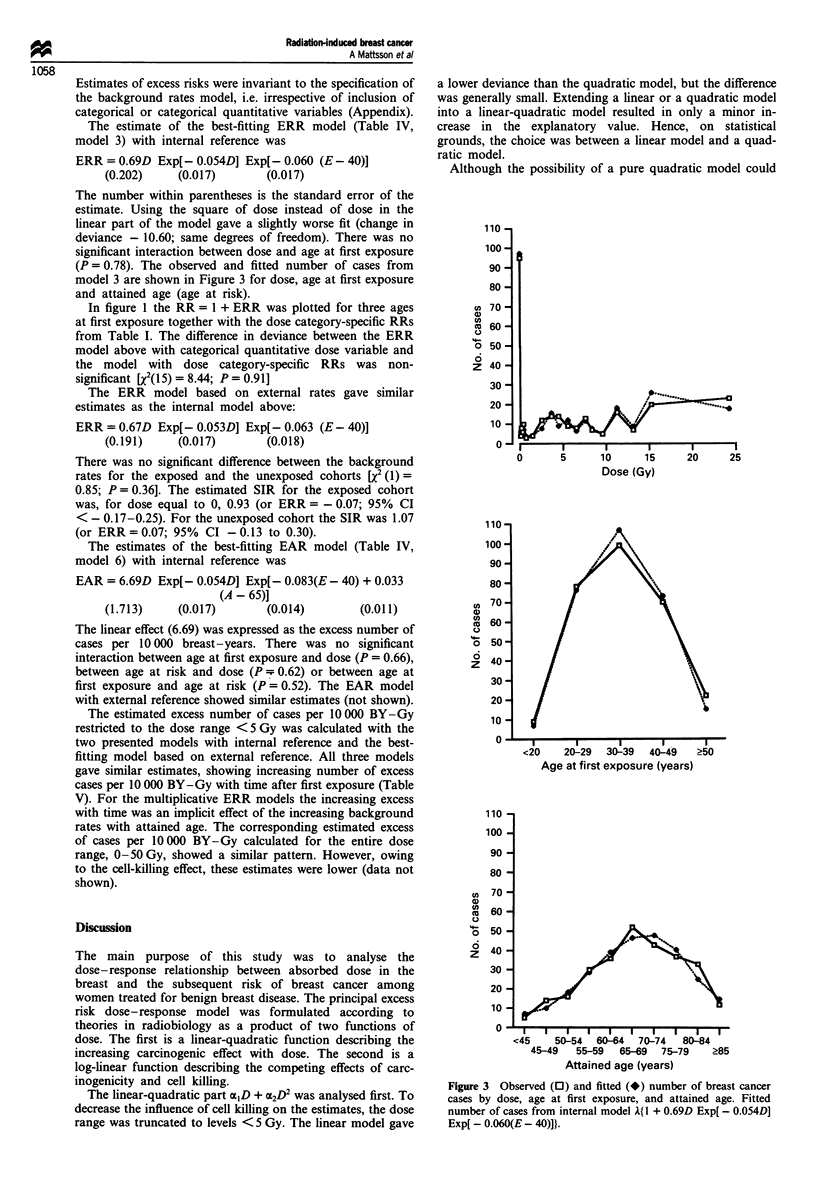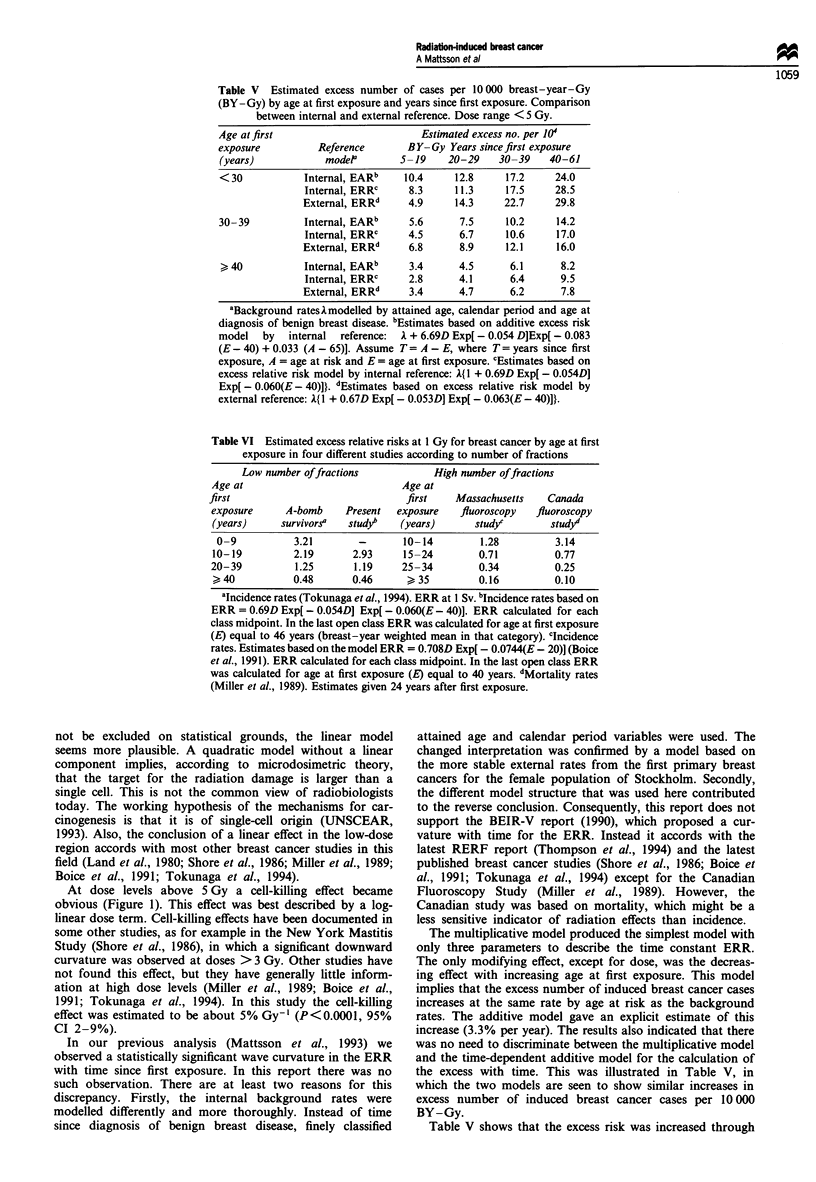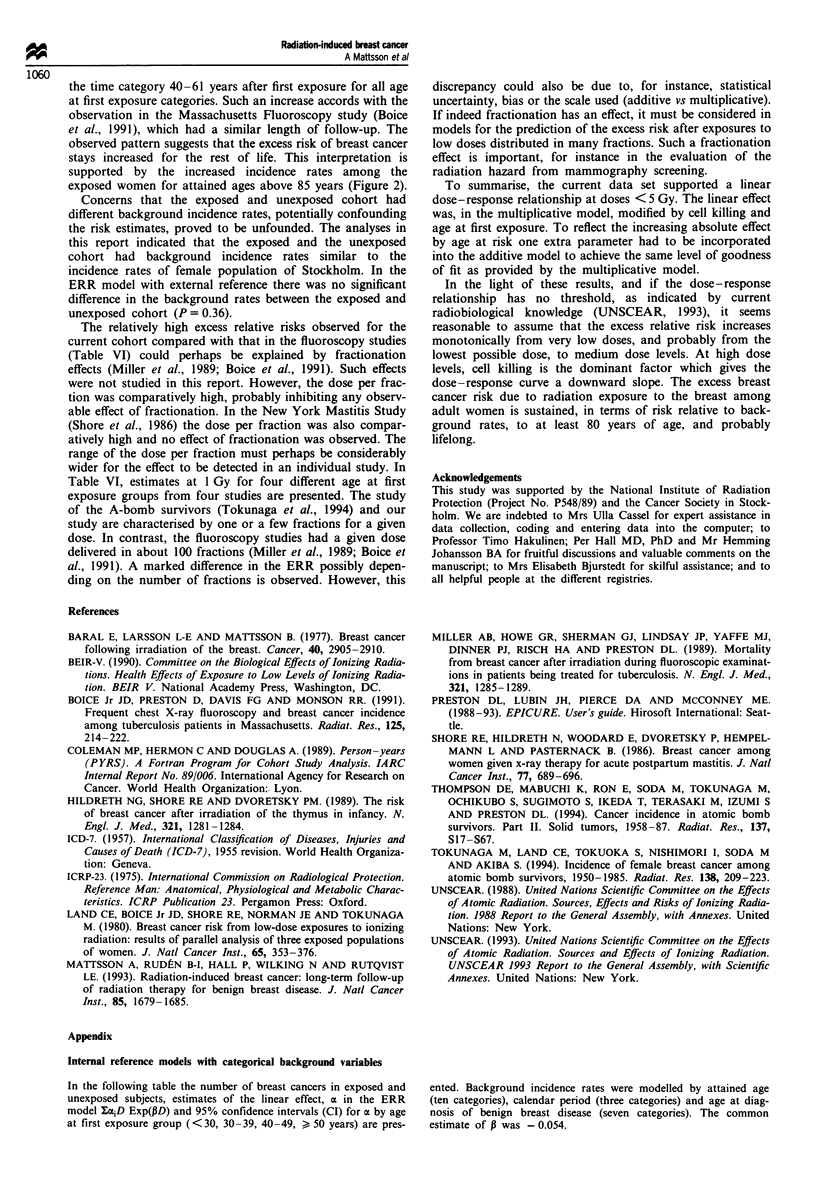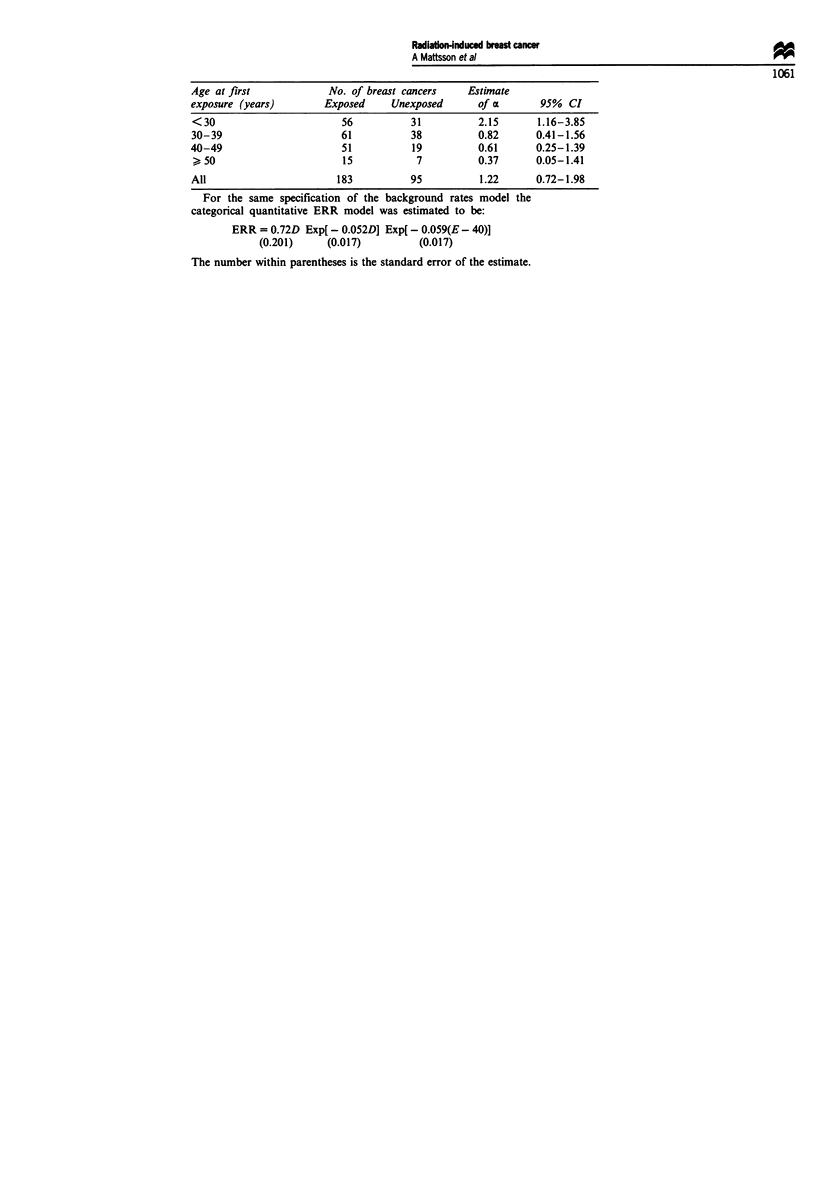# Dose- and time-response for breast cancer risk after radiation therapy for benign breast disease.

**DOI:** 10.1038/bjc.1995.461

**Published:** 1995-10

**Authors:** A. Mattsson, B. I. Rudén, J. Palmgren, L. E. Rutqvist

**Affiliations:** Oncologic Centre, Karolinska Hospital, Stockholm, Sweden.

## Abstract

Exposure of the breast to ionising radiation increases the risk of breast cancer, especially among young women. However, some issues remain controversial, for instance the shape of the dose-response curve and the expression of time-related excess. The main purpose of this report was to examine the dose-response curves for radiation-induced breast cancer formulated according to radiobiological target theories. Another purpose was to analyse the time-related excess of breast cancer risk after exposure when dose and age at first exposure were held constant. Breast cancer incidence was analysed in a cohort of 3090 women diagnosed with benign breast disease during 1925-61 (median age 37 years). Of these, 1216 were treated with radiation therapy. The dose range was 0-50 Gy (mean 5.8 Gy). The incidence rate as function of dose was analysed using a linear-quadratic Poisson regression model. Cell-killing effects and other modifying effects were incorporated through additional log-linear terms. Additive and multiplicative models were compared in estimating the time-related excess. The analysis, which was based on 278 breast cancer cases, showed a linear dose-response relationship at low to medium dose levels with a cell-killing effect of 5% Gy-1 (95% confidence interval 2-9%). For a given absorbed dose and age at first exposure the time-related excess was proportional to the background rates with a suggestion that the excess remains throughout life.


British Journal of Cancer (1995) 72, 1054-1061
© 1995 Stockton Press All rights reserved 0007-0920/95 $12.00

### Dose- and time-response for breast cancer risk after radiation therapy for benign breast disease

A Mattsson[1], BI Rudén[2], J Palmgren[3] and LE Rutqvist[1]

[1]*Oncologic Centre,* [2]*Department of Hospital Physics and* [3]*Department of Cancer Epidemiology, Karolinska Hospital, PO Box 100, S-171 76 Stockholm, Sweden.*

**Summary** Exposure of the breast to ionising radiation increases the risk of breast cancer, especially among young women. However, some issues remain controversial, for instance the shape of the dose-response curve and the expression of time-related excess. The main purpose of this report was to examine the dose-response curves for radiation-induced breast cancer formulated according to radiobiological target theories. Another purpose was to analyse the time-related excess of breast cancer risk after exposure when dose and age at first exposure were held constant. Breast cancer incidence was analysed in a cohort of 3090 women diagnosed with benign breast disease during 1925-61 (median age 37 years). Of these, 1216 were treated with radiation therapy. The dose range was 0-50 Gy (mean 5.8 Gy). The incidence rate as function of dose was analysed using a linear-quadratic Poisson regression model. Cell-killing effects and other modifying effects were incorporated through additional log-linear terms. Additive and multiplicative models were compared in estimating the time-related excess. The analysis, which was based on 278 breast cancer cases, showed a linear dose-response relationship at low to medium dose levels with a cell-killing effect of 5% $Gy^{-1}$ (95% confidence interval 2-9%). For a given absorbed dose and age at first exposure the time-related excess was proportional to the background rates with a suggestion that the excess remains throughout life.

**Keywords:** ionising radiation; breast cancer; cohort study; dose-response; time-response

It is well known that exposure of the breast to ionising radiation increases the risk of subsequent breast cancer, especially among young women (Baral *et al.*, 1977; Land *et al.*, 1980; Shore *et al.*, 1986; Hildreth *et al.*, 1989; Miller *et al.*, 1989; Boice *et al.*, 1991; Tokunaga *et al.*, 1994). According to radiobiological target theories, the cancer risk is expected to increase approximately linearly at low doses with an upward curvature at medium dose levels (UNSCEAR, 1993). Simultaneously there is a potential competing effect of cell killing that should negatively modify the response at high dose levels (UNSCEAR, 1993).

The main purpose of this report is to present statistical analyses of models for dose-response curves of the mentioned type. The data were from a previously described cohort study (Mattsson *et al.*, 1993). The cohort consisted of 3090 women who presented with clinical signs and symptoms of benign breast disease from the 1920s until the 1950s. Of these, 1216 were treated with radiotherapy. The absorbed dose ranged from 0 to about 50 Gy.

When estimating the lifetime excess risk of radiation-induced breast cancer it is important to determine the risk pattern over time since exposure and/or by attained age (UNSCEAR, 1988; BEIR-V, 1990). Another purpose of this report is, therefore, to describe the excess risk over time. The analyses were done for a given dose and age at first exposure pattern. Excess additive risk and excess relative risk models with and without time-varying risk estimates were compared. In models without time variation the additive model produces excess *absolute* risks that are constant irrespective of the background rates, whereas the relative risk model produces excess risks that are proportional (relative) to the background rates.

Understanding dose and time patterns as well as dependence on age at first exposure is important in evaluations of the benefit of mammographic screening or of post-operative radiation therapy for early-stage breast cancer.

Correspondence: A Mattsson
Received 24 November 1994; revised 10 April 1995; accepted 22 May 1995

### Materials and methods

### *Materials*

The patients, methods of follow-up, radiation techniques, methods of determination of absorbed dose, absorbed doses and results focusing on age at first exposure and time since first exposure were published in a previous report (Mattsson *et al.*, 1993). The cohort consisted of 1216 exposed and 1874 unexposed women who presented with clinical signs/symptoms of benign breast disease during the period 1925-61. The clinical diagnoses were fibroadenomatosis (93%), acute mastitis (4%) or chronic mastitis (3%). The women had no previous history of breast cancer and there was no suspicion of breast cancer or a precancerous lesion at the time of diagnosis. For instance, women with bloody discharge from the nipple or a history of atypical epithelial proliferation at biopsy were excluded. Patients were also excluded if the available follow-up information showed that they were diagnosed with breast cancer before 1958. The exposed women were irradiated at the Department of Radiotherapy, Karolinska Hospital. The unexposed women had been referred to that same institution, but had not received radiation therapy. The median age at first exposure and/or diagnosis was 40 years (range 8-74 years) for the exposed patients and 36 years (range 10-78 years) for the unexposed.

To determine end point data in the cohort, the computerised files were linked with the Swedish Cancer Register to obtain the breast cancer cases, the Swedish Cause of Death Register to obtain the dates of death and the National Population Register to obtain the dates of emigration.

To determine the absorbed dose to each individual breast, the radiation therapy was simulated according to the original treatment charts using a Randophantom. Thermoluminescent dosimetry lithium fluoride (LiF) discs were placed in the phantom and radiation was given according to the different treatment techniques that had been used according to the patients' records. The accuracy of the determined absorbed dose in the Randophantom was ± 10%. As the medical records did not provide information about the size of the breast, an average size was estimated from ICRP-23 (1975).

In 83% of the treated patients in whom the whole breast was irradiated, a tangential technique with opposed beams was used, with an angulation of the beams from the horizontal direction. When only a part of the breast was treated (17% of the treated patients), the beam was medial or lateral to the breast or perpendicular to the chest wall. For these breasts the mean absorbed dose was calculated by the multiplication of the average absorbed dose in the primary irradiated volume and the ratio of the primary irradiated volume to the total volume of the breast.

The average total mean absorbed dose to 2432 exposed breasts was 5.84 Gy (range 0.003–50.14 Gy). The lowest values relate to the contralateral breast in patients who only received treatment to the axilla.

In the previously published study (Mattsson *et al.*, 1993) the overall radiation-associated relative risk (RR) was 3.58 [95% confidence interval (CI) 2.77–4.63]. There was a positive log-linear association between dose and RR with a levelling off at high doses. The RR decreased with increased age at first exposure, but was still increased at ages above 40 years. The effect over time showed a wave-like pattern with a maximum at about 25 years after first exposure.

There was no statistically significant differences in the RR of subsequent breast cancer between women with different clinical diagnoses of benign breast disease. An age- and calendar time-adjusted RR of 1.16 for untreated breasts among the exposed cohort (mean dose 0.27 Gy) *vs* breasts in the unexposed cohort indicated that the two cohorts probably had roughly the same background risk. The time between the first and last treatment–adjusted for dose–did not significantly influence the RR.

This report contains, in contrast to the previous report (Mattsson *et al.*, 1993), results from analyses of the dose–response relationship according to radiobiological models. Moreover, a comparison between models with internal and external reference rates was done to scrutinise the earlier proposed time wave (Mattsson *et al.*, 1993). Intrinsic to the time wave issue is the comparison of which of the two models–additive, or relative risk–best describes the time excess of breast cancer after exposure of the breast to ionising radiation.

### *Statistical methods*

All analyses in this report were restricted to the first primary breast cancer. Of the three synchronously diagnosed bilateral breast cancer cases, one was randomly chosen to represent the first primary breast cancer. It was common that the absorbed dose in the left and right breasts of an individual differed considerably. To permit meaningful dose–response analyses, the breast was, therefore, defined as the carrier of risk. Breast–years were calculated from 1 January 1958, or from date of diagnosis of benign breast disease for those diagnosed later, to date of diagnosis of breast cancer, to date of death, emigration or to 31 December 1987. The contralateral breast was censored at the date of diagnosis of the first primary breast cancer. Data covering the first 5 years after diagnosis of benign breast disease were not included in the analyses to avoid inclusion of preclinical cases of breast cancer. The program PYRS (Coleman *et al.*, 1989) was used for the breast–year calculation.

Information about the number of breast cancer cases during the period from 1958 to 1987 was obtained by linking the computer files with the Swedish Cancer Register. This register was established on 1 January 1958. Each new primary tumour is recorded according to the ICD-7 (1957) as a separate case. Data indicating tumour laterality were obtained from the original notifications (filed at the Swedish Cancer Registry) for cases that occurred before 1970. Thereafter, such data were available from the registry in computerised form.

Analyses were done with both internal and external incidence rates. The external rates were from the female population in Stockholm, since most of the patients were residents of that region. Models based on external rates were primarily used to evaluate the stability of the internal background rates, especially over time since first exposure. The expected numbers of cases, which were used in the external analyses, were calculated by multiplying age- and calendar year-specific breast–years by the breast-specific incidence rates and then summed. The breast-specific incidence rates were based on the first diagnosed primary breast cancer for each affected woman in the Stockholm population. However, such rates can only be computed based on computerised data for the period 1970 to the present. For the period 1958–69 breast-specific incidence rates had to be estimated. This was done assuming that the age- and breast-specific relative distributions between left and right breast during 1958–69 were identical to the corresponding distributions for the period 1970–87. The standardised incidence ratio (SIR) was defined as observed over expected number of cases.

Most inferences were by excess relative risk (ERR) models of the form:

$$\lambda_{A,C}\lambda_B \ \{1 + f_1(D) \ \mathrm{Exp}[f_2(D)] \ \mathrm{Exp}[f_3(E,\ T,\ A,)]\}$$

The background rates $\lambda$ were in all analyses classified for $A$ = attained age during follow-up (ten categories), $C$ = calendar period (three categories) and $B$ = age at diagnosis of benign breast disease (seven categories). $\lambda_{A,C}$ values were in the external analyses the incidence rates of the female population of Stockholm. In the internal analyses the background incidence rates were estimated within the cohort itself. The term $\lambda_B$ accounts for the dependence of background rates on age at diagnosis of benign breast disease. The linear-quadratic function $f_1(D)$ was generally formulated as $\alpha_1 D + \alpha_2 D^2$, where $D$ denotes total mean absorbed dose (referred to as dose) to the breast. The dose variable was categorical quantitative and treated as a continuous variable. The assigned values were the mean doses from the 19 categories given in Table I. In the aim of estimating an SIR for the exposed cohort and dose =O, a cohort indicator variable was incorporated in the linear part of the external model. The first log-linear term, $f_2(D)$, was used to model the potential modification of cell killing on the effect estimated by $f_1(D)$. The function $f_2$ could potentially comprise $D$ and/or $D^2$ (dose, defined as above). The second log-linear term $f_3(E, T, A)$, was incorporated in the model to take account of dose effect modifiers. This means that the shape of the dose–response relationship is taken to apply when the variables in $f_3$ are held constant. $E$ was age at first exposure with seven categories, $T$ time since first exposure with seven categories and $A$ attained age (i.e. age at risk) with ten categories. $T$ and $A$ were always incorporated after $E$ and were therefore not allowed to be in the same model depending on the restriction $A = E + T$. When $E$, $T$ and $A$ were coded as categorical quantitative variables, the mean values were assigned to the different categories.

In one model, test for curvature in the ERR with time since first exposure was tested by the term $\ln^2(T/25)$ in $f_3$ with $T$ categorical quantitative. This non-linear form was chosen because it had been used to describe the time dependence of the RR in previously published reports on radiation-induced breast cancer (BEIR-V, 1990; Mattson *et al.*, 1993).

Analogous excess additive risk (EAR) models of the form

$$\lambda_{A,C}\lambda_B + f_1(D) \ \mathrm{Exp}[f_2(D)] \ \mathrm{Exp}[f_3(E,\ T,\ A)]$$

were also fitted and compared with ERR models. The EAR was expressed as the number of excess cases per 10 000 breast–years.

When the EAR and ERR models were compared, the background rates were modelled both by categorical variables and by categorical quantitative variables. When age at diagnosis of benign breast disease and calendar period were formulated as categorical quantitative variables, the assigned values were consecutive integers from 1 onward. Categorical quantitative attained age was assigned values as above. Risk estimates for categorical quantitative background rates models are presented in the results section. For comparison

**Table I** Number of breast-years, number of breast cancer cases, and relative risk (RR) by mean absorbed dose to the breast

| *Dose (Gy)* | *Mean dose (Gy)* | *Breast-years* | *Cases* | *RR*[a] |
|---|---|---|---|---|
| 0 | 0 | 92,785 | 95 | 1.00 |
| 0.003-0.09 | 0.065 | 5,095 | 4 | 0.69 |
| 0.10-0.19 | 0.149 | 4,614 | 8 | 1.49 |
| 0.20-0.34 | 0.276 | 5,334 | 6 | 1.13 |
| 0.35-0.49 | 0.392 | 5,613 | 10 | 2.08 |
| 0.50-0.99 | 0.684 | 2,320 | 3 | 1.40 |
| 1.00-1.99 | 1.423 | 1,394 | 4 | 2.62 |
| 2.00-2.99 | 2.487 | 1,878 | 12 | 5.66 |
| 3.00-3.99 | 3.628 | 2,781 | 14 | 4.03 |
| 4.00-4.99 | 4.451 | 1,614 | 14 | 7.39 |
| 5.00-5.99 | 5.535 | 1,770 | 9 | 4.24 |
| 6.00-6.99 | 6.520 | 1,004 | 8 | 7.45 |
| 7.00-7.99 | 7.584 | 1,417 | 13 | 7.31 |
| 8.00-8.99 | 8.352 | 1,120 | 7 | 5.95 |
| 9.00-9.99 | 9.569 | 817 | 5 | 6.25 |
| 10.00-11.99 | 11.250 | 2,929 | 16 | 5.62 |
| 12.00-13.99 | 13.121 | 1,404 | 7 | 5.29 |
| 14.00-15.99 | 15.266 | 4,973 | 20 | 4.75 |
| ⩾ 16.00 | 24.239 | 2,893 | 23 | 8.81 |
| Total exposure | 5.840 | 48,970 | 183 | 3.53 |

[a]Background incidence rates modelled by categorical quantitative attained age, calendar period and age at diagnosis of benign breast disease.

**Table II** Comparison between linear, quadratic and linear-quadratic excess relative risk models and the saturated excess relative risk model[a] for two different truncations of dose. Internal reference

| | | *Dose truncation: <3 Gy* | | | | *Dose truncation: <5 Gy* | | |
|---|---|---|---|---|---|---|---|---|
| *Model no.* | *Deviance* | *Difference in deviance* | *Difference in d.f.* | *$\chi^2$-based* P-*value* | *Deviance* | *Difference in deviance* | *Difference in d.f.* | *$\chi^2$-based* P-*value* |
| 1. Saturated model | 496.64 | – | – | – | 624.89 | – | – | – |
| 2. $1+\alpha_{11}D$ | 499.74 | 3.10 | 6 | 0.80 | 630.44 | 5.55 | 8 | 0.70 |
| 3. $1+\alpha_{21}D^2$ | 500.01 | 3.37 | 6 | 0.76 | 635.31 | 10.42 | 8 | 0.24 |
| 4. $1+\alpha_{12}D+\alpha_{22}D^2$ | 499.13 | 2.49 | 5 | 0.78 | 630.35 | 5.45 | 7 | 0.60 |

[a]Background incidence rates modelled by categorical quantitative attained age, calendar period and age at diagnosis of benign breast disease. Saturated model dose categories as in Table I.

estimates from categorical background rates models are presented in the Appendix.

For an evaluation of the linear-quadratic form $f_1(D) = \alpha_1 D + \alpha_2 D^2$, the dose range was constrained to the subset <5 Gy. This was done to reduce the effects of cell killing on the estimates of $\alpha_1$ and $\alpha_2$. The form of the dose–response relationship was analysed for two truncated dose intervals, <3 Gy and <5 Gy. The best-fitting form of $f(D)$ in the subset <5 Gy was then assumed to apply when models incorporating the log-linear terms were fitted to the unconstrained dose range 0–50.14 Gy.

Poisson regression models were fitted using the program AMFIT (Preston *et al.*, 1988–93). Estimation of parameters was done by maximum-likelihood methods. Differences in deviance, a measure of unexplained variability, were used to compare nested Poisson regression models. Change in deviance between two nested models is approximately $\chi^2$ distributed, with degrees of freedom equal to the difference in the number of parameters in the two models. Confidence intervals were computed by likelihood-based methods.

The number of breast-year-Gy (BY-Gy) was calculated as the number of breast-years times the mean absorbed dose for the different cells of the frequency table to which the models were fitted. The estimated number of excess cases per 10 000 BY-Gy was calculated as a ratio of two sums: the sum of the differences between the number of fitted cases and the number of fitted background cases and the sum of the number of 10 000 BY-Gy.

### Results

The total number of observed breast cancer cases was 278, of which 95 were in the unexposed cohort. In the analyses of the dose–response relationship for doses <5 Gy, 75 cases were from the exposed cohort, of which 47 were exposed to <3 Gy (Table I). For both these truncation points the model linear in dose provided the best fit (Table II). There was no loss of fit when the linear model was compared with a saturated model for categorised dose. For the internal reference ERR model, the estimate of $\alpha_{11}$, the linear effect of a unit change of dose (in Gy) was 1.63 (95% CI 0.77–2.89) and 1.31 (95% CI 0.79–2.04) for the <3 Gy and <5 Gy truncation respectively. The estimates were insensitive to the use of internal or external incidence rates (data not shown).

### *Cell killing*

As shown in Figure 1, the increase in the RR levelled off at high doses. This effect was best described by a log-linear term with an effect estimate of 5% $\text{Gy}^{-1}$ ($P<0.0001$; 95% CI 2–9%). Other tested possibilities included a log-quadratic term and a log-linear-quadratic term. The fit with a log-quadratic term was slightly worse with the same degrees of freedom, compared with the fit with a log-linear term. The log-linear-quadratic term did not give meaningful estimates owing to collinearity. The estimate of the corresponding parameter correlation coefficient was −0.97.

### *Age at first exposure*

The estimated ERR per Gy decreased with increasing age at first exposure (Appendix). Without loss of fit this decrease could be formulated as a log-linear trend [$\chi^2(5) = 6.3$; $P = 0.28$] with an estimate of −6% per year of increased age at first exposure (95% CI −10% to −2%).

### *The time pattern of the excess risk*

The crude age-specific incidence rates for the unexposed cohort followed closely the incidence rates for the female population of Stockholm (Figure 2). The estimated increases

**Figure 1** Dose–response curves: dose category-specific RR from Table I and fitted RR for breast cancer for three ages at first exposure from internal model $RR = 1 + 0.69D\ \text{Exp}[-0.054D]\ \text{Exp}[-0.060(E-40)]$.

**Figure 2** Logarithm of crude breast cancer incidence rate per 10 000 breast-years by attained age for exposed and unexposed cohorts, and Stockholm female population 1958–87.

**Table III** Test for heterogeneity with time since first exposure within three age at first exposure groups. The test was done with ERR model[a] $\Sigma\alpha_t D\ \text{Exp}\{\beta D\}$ where *t* is time since first exposure interval

| *Age at first exposure (years)* | *Reference* | *Time since first exposure (years)* 5–19 $\alpha_1$ est. | 20–29 $\alpha_2$ est. | 30–39 $\alpha_3$ est. | ≥40 $\alpha_4$ est. | *Test for heterogeneity* $\chi^2$ (3) | (P) | *Test for curvature*[b] $\chi^2$ (1) | (P) |
|---|---|---|---|---|---|---|---|---|---|
| <30 | Internal | 4.44 | 3.14 | 1.91 | 1.78 | 1.39 | (0.71) | 0.003 | (0.96) |
| | External | 3.61 | 2.33 | 2.19 | 2.75 | 0.62 | (0.89) | 1.02 | (0.31) |
| 30–39 | Internal | 0.91 | 1.56 | 1.03 | 0.22 | 6.71 | (0.08) | 5.96 | (0.015) |
| | External | 0.58 | 0.63 | 0.76 | 0.58 | 0.42 | (0.94) | 0.20 | (0.66) |
| ≥40 | Internal | 0.29 | 0.47 | 0.36 | 0.53 | 0.70 | (0.87) | 1.36 | (0.24) |
| | External | 0.16 | 0.38 | 0.30 | 0.44 | 2.36 | (0.50) | 2.32 | (0.13) |

[a]Background incidence rates were classified on attained age, calendar period and age at diagnosis of benign breast disease. [b]Test function: $W = \ln^2(T/25)$ in ERR model $\alpha D\ \text{Exp}\{\beta D\}\ \text{Exp}\{\tau W\}$. *T* was categorical quantitative.

**Table IV** Comparison between fit of internal reference multiplicative model and internal reference additive model

| *Variables*[a] *Model no.* | *Multiplicative (ERR) model* *Deviance* | *Difference in deviance* | *Difference in df* | $\chi^2$*-based* P*-value* | *Additive (EAR) model* *Deviance* | *Difference in deviance* | *Difference in df* | $\chi^2$*-based* P*-value* |
|---|---|---|---|---|---|---|---|---|
| *Background* $(\lambda)^b$ | 1334.34 | – | – | – | 1334.34 | – | – | – |
| *Linear term,* $f_1$ (.) | | | | | | | | |
| 1.*D* | 1183.28 | 151.06 | 1 | <0.0001 | 1206.48 | 127.86 | 1 | <0.0001 |
| *Log-linear term,* $f_2$ (.) | | | | | | | | |
| 2. Model 1 +*D* | 1164.64 | 18.64 | 1 | <0.0001 | 1181.99 | 24.49 | 1 | <0.0001 |
| *Log-linear term,* $f_3$ (.) | | | | | | | | |
| 3. Model 2 +*E* | 1154.20 | 10.44 | 1 | 0.001 | 1163.00 | 18.99 | 1 | <0.0001 |
| 4. Model 3 +$T_{cat}$ | 1149.40 | 4.80 | 3 | 0.187 | 1157.31 | 5.69 | 3 | 0.128 |
| 5. Model 3 +$A_{cat}$ | 1152.64 | 1.56 | 3 | 0.668 | 1151.36 | 11.64 | 3 | 0.009 |
| 6. Model 3 +*A* | | | | | 1154.22 | 8.78 | 1 | 0.003 |

[a]D, absorbed dose; *E*, age at first exposure, categorical quantitative; $T_{cat}$, categorical time since first exposure (5–19, 20–29, 30–39 and ≥40 years); $A_{cat}$, categorical attained age (<55, 55–64, 65–74, ≥75 years); A, categorical quantitative age at risk centred to 65 years of age. [b]Background rates (λ) modelled by categorical quantitative attained age, calendar period and age at diagnosis of benign breast disease.

per year of attained age were 3.0% and 3.3% respectively. No significant difference in the increase between the exposed and unexposed women was observed ($P = 0.38$), indicating a time-constant multiplicative effect of exposure on the background rates. Similarly, when the ERR with time was analysed in the model, holding dose and age at first exposure constant, no persistent heterogeneity or curvature was observed (Table III). A statistically significant curvature was only observed in the 30–39 year age at first exposure group when using the internal reference. However, the curvature disappeared when the external reference was used.

### *ERR and EAR models*

In a comparison between the multiplicative (ERR) model and the additive (EAR) model, both with internal reference, the former gave a slightly more parsimonious description of the data (Table IV). Model 3, with dose and age at first exposure, was the best-fitting multiplicative model. Neither the general test for a modifying effect of time since first exposure nor age at risk was significant ($P = 0.19$ and $P = 0.67$ respectively). For additive risk, model 6 with dose, age at first exposure and age at risk gave the best fit.

Estimates of excess risks were invariant to the specification of the background rates model, i.e. irrespective of inclusion of categorical or categorical quantitative variables (Appendix).

The estimate of the best-fitting ERR model (Table IV, model 3) with internal reference was

$$\text{ERR} = \underset{(0.202)}{0.69D}\ \text{Exp}[-\underset{(0.017)}{0.054D}]\ \text{Exp}[-\underset{(0.017)}{0.060}\ (E-40)]$$

The number within parentheses is the standard error of the estimate. Using the square of dose instead of dose in the linear part of the model gave a slightly worse fit (change in deviance − 10.60; same degrees of freedom). There was no significant interaction between dose and age at first exposure ($P = 0.78$). The observed and fitted number of cases from model 3 are shown in Figure 3 for dose, age at first exposure and attained age (age at risk).

In figure 1 the RR = 1 + ERR was plotted for three ages at first exposure together with the dose category-specific RRs from Table I. The difference in deviance between the ERR model above with categorical quantitative dose variable and the model with dose category-specific RRs was non-significant [$\chi^2(15) = 8.44$; $P = 0.91$]

The ERR model based on external rates gave similar estimates as the internal model above:

$$\text{ERR} = \underset{(0.191)}{0.67D}\ \text{Exp}[-\underset{(0.017)}{0.053D}]\ \text{Exp}[-\underset{(0.018)}{0.063}\ (E-40)]$$

There was no significant difference between the background rates for the exposed and the unexposed cohorts [$\chi^2(1) = 0.85$; $P = 0.36$]. The estimated SIR for the exposed cohort was, for dose equal to 0, 0.93 (or ERR = − 0.07; 95% CI < − 0.17–0.25). For the unexposed cohort the SIR was 1.07 (or ERR = 0.07; 95% CI − 0.13 to 0.30).

The estimates of the best-fitting EAR model (Table IV, model 6) with internal reference was

$$\text{EAR} = \underset{(1.713)}{6.69D}\ \text{Exp}[-\underset{(0.017)}{0.054D}]\ \text{Exp}[-\underset{(0.014)}{0.083}(E-40) + \underset{(0.011)}{0.033}\ (A-65)]$$

The linear effect (6.69) was expressed as the excess number of cases per 10 000 breast-years. There was no significant interaction between age at first exposure and dose ($P = 0.66$), between age at risk and dose ($P \mp 0.62$) or between age at first exposure and age at risk ($P = 0.52$). The EAR model with external reference showed similar estimates (not shown).

The estimated excess number of cases per 10 000 BY–Gy restricted to the dose range < 5 Gy was calculated with the two presented models with internal reference and the best-fitting model based on external reference. All three models gave similar estimates, showing increasing number of excess cases per 10 000 BY–Gy with time after first exposure (Table V). For the multiplicative ERR models the increasing excess with time was an implicit effect of the increasing background rates with attained age. The corresponding estimated excess of cases per 10 000 BY–Gy calculated for the entire dose range, 0–50 Gy, showed a similar pattern. However, owing to the cell-killing effect, these estimates were lower (data not shown).

### Discussion

The main purpose of this study was to analyse the dose–response relationship between absorbed dose in the breast and the subsequent risk of breast cancer among women treated for benign breast disease. The principal excess risk dose–response model was formulated according to theories in radiobiology as a product of two functions of dose. The first is a linear-quadratic function describing the increasing carcinogenic effect with dose. The second is a log-linear function describing the competing effects of carcinogenicity and cell killing.

The linear-quadratic part $\alpha_1 D + \alpha_2 D^2$ was analysed first. To decrease the influence of cell killing on the estimates, the dose range was truncated to levels < 5 Gy. The linear model gave a lower deviance than the quadratic model, but the difference was generally small. Extending a linear or a quadratic model into a linear-quadratic model resulted in only a minor increase in the explanatory value. Hence, on statistical grounds, the choice was between a linear model and a quadratic model.

Although the possibility of a pure quadratic model could

**Figure 3** Observed (□) and fitted (◆) number of breast cancer cases by dose, age at first exposure, and attained age. Fitted number of cases from internal model $\lambda\{1 + 0.69D\ \text{Exp}[-0.054D]\ \text{Exp}[-0.060(E-40)]\}$.

**Table V** Estimated excess number of cases per 10 000 breast-year-Gy (BY-Gy) by age at first exposure and years since first exposure. Comparison between internal and external reference. Dose range <5 Gy.

| *Age at first exposure (years)* | *Reference model*[a] | *Estimated excess no. per $10^4$ BY-Gy Years since first exposure* 5-19 | 20-29 | 30-39 | 40-61 |
|---|---|---|---|---|---|
| <30 | Internal, EAR[b] | 10.4 | 12.8 | 17.2 | 24.0 |
| | Internal, ERR[c] | 8.3 | 11.3 | 17.5 | 28.5 |
| | External, ERR[d] | 4.9 | 14.3 | 22.7 | 29.8 |
| 30-39 | Internal, EAR[b] | 5.6 | 7.5 | 10.2 | 14.2 |
| | Internal, ERR[c] | 4.5 | 6.7 | 10.6 | 17.0 |
| | External, ERR[d] | 6.8 | 8.9 | 12.1 | 16.0 |
| ⩾40 | Internal, EAR[b] | 3.4 | 4.5 | 6.1 | 8.2 |
| | Internal, ERR[c] | 2.8 | 4.1 | 6.4 | 9.5 |
| | External, ERR[d] | 3.4 | 4.7 | 6.2 | 7.8 |

[a]Background rates $\lambda$ modelled by attained age, calendar period and age at diagnosis of benign breast disease. [b]Estimates based on additive excess risk model by internal reference: $\lambda + 6.69D\ \mathrm{Exp}[-0.054\ D]\mathrm{Exp}[-0.083\ (E-40) + 0.033\ (A-65)]$. Assume $T = A - E$, where $T$ = years since first exposure, $A$ = age at risk and $E$ = age at first exposure. [c]Estimates based on excess relative risk model by internal reference: $\lambda\{1 + 0.69D\ \mathrm{Exp}[-0.054D]\ \mathrm{Exp}[-0.060(E-40)]\}$. [d]Estimates based on excess relative risk model by external reference: $\lambda\{1 + 0.67D\ \mathrm{Exp}[-0.053D]\ \mathrm{Exp}[-0.063(E-40)]\}$.

**Table VI** Estimated excess relative risks at 1 Gy for breast cancer by age at first exposure in four different studies according to number of fractions

| *Low number of fractions* *Age at first exposure (years)* | *A-bomb survivors*[a] | *Present study*[b] | *High number of fractions* *Age at first exposure (years)* | *Massachusetts fluoroscopy study*[c] | *Canada fluoroscopy study*[d] |
|---|---|---|---|---|---|
| 0-9 | 3.21 | – | 10-14 | 1.28 | 3.14 |
| 10-19 | 2.19 | 2.93 | 15-24 | 0.71 | 0.77 |
| 20-39 | 1.25 | 1.19 | 25-34 | 0.34 | 0.25 |
| ⩾40 | 0.48 | 0.46 | ⩾35 | 0.16 | 0.10 |

[a]Incidence rates (Tokunaga *et al.*, 1994). ERR at 1 Sv. [b]Incidence rates based on ERR = $0.69D\ \mathrm{Exp}[-0.054D]\ \mathrm{Exp}[-0.060(E-40)]$. ERR calculated for each class midpoint. In the last open class ERR was calculated for age at first exposure ($E$) equal to 46 years (breast-year weighted mean in that category). [c]Incidence rates. Estimates based on the model ERR = $0.708D\ \mathrm{Exp}[-0.0744(E-20)]$ (Boice *et al.*, 1991). ERR calculated for each class midpoint. In the last open class ERR was calculated for age at first exposure ($E$) equal to 40 years. [d]Mortality rates (Miller *et al.*, 1989). Estimates given 24 years after first exposure.

not be excluded on statistical grounds, the linear model seems more plausible. A quadratic model without a linear component implies, according to microdosimetric theory, that the target for the radiation damage is larger than a single cell. This is not the common view of radiobiologists today. The working hypothesis of the mechanisms for carcinogenesis is that it is of single-cell origin (UNSCEAR, 1993). Also, the conclusion of a linear effect in the low-dose region accords with most other breast cancer studies in this field (Land *et al.*, 1980; Shore *et al.*, 1986; Miller *et al.*, 1989; Boice *et al.*, 1991; Tokunaga *et al.*, 1994).

At dose levels above 5 Gy a cell-killing effect became obvious (Figure 1). This effect was best described by a log-linear dose term. Cell-killing effects have been documented in some other studies, as for example in the New York Mastitis Study (Shore *et al.*, 1986), in which a significant downward curvature was observed at doses >3 Gy. Other studies have not found this effect, but they have generally little information at high dose levels (Miller *et al.*, 1989; Boice *et al.*, 1991; Tokunaga *et al.*, 1994). In this study the cell-killing effect was estimated to be about 5% $\mathrm{Gy}^{-1}$ ($P<0.0001$, 95% CI 2-9%).

In our previous analysis (Mattsson *et al.*, 1993) we observed a statistically significant wave curvature in the ERR with time since first exposure. In this report there was no such observation. There are at least two reasons for this discrepancy. Firstly, the internal background rates were modelled differently and more thoroughly. Instead of time since diagnosis of benign breast disease, finely classified attained age and calendar period variables were used. The changed interpretation was confirmed by a model based on the more stable external rates from the first primary breast cancers for the female population of Stockholm. Secondly, the different model structure that was used here contributed to the reverse conclusion. Consequently, this report does not support the BEIR-V report (1990), which proposed a curvature with time for the ERR. Instead it accords with the latest RERF report (Thompson *et al.*, 1994) and the latest published breast cancer studies (Shore *et al.*, 1986; Boice *et al.*, 1991; Tokunaga *et al.*, 1994) except for the Canadian Fluoroscopy Study (Miller *et al.*, 1989). However, the Canadian study was based on mortality, which might be a less sensitive indicator of radiation effects than incidence.

The multiplicative model produced the simplest model with only three parameters to describe the time constant ERR. The only modifying effect, except for dose, was the decreasing effect with increasing age at first exposure. This model implies that the excess number of induced breast cancer cases increases at the same rate by age at risk as the background rates. The additive model gave an explicit estimate of this increase (3.3% per year). The results also indicated that there was no need to discriminate between the multiplicative model and the time-dependent additive model for the calculation of the excess with time. This was illustrated in Table V, in which the two models are seen to show similar increases in excess number of induced breast cancer cases per 10 000 BY-Gy.

Table V shows that the excess risk was increased through the time category 40–61 years after first exposure for all age at first exposure categories. Such an increase accords with the observation in the Massachusetts Fluoroscopy study (Boice *et al.*, 1991), which had a similar length of follow-up. The observed pattern suggests that the excess risk of breast cancer stays increased for the rest of life. This interpretation is supported by the increased incidence rates among the exposed women for attained ages above 85 years (Figure 2).

Concerns that the exposed and unexposed cohort had different background incidence rates, potentially confounding the risk estimates, proved to be unfounded. The analyses in this report indicated that the exposed and the unexposed cohort had background incidence rates similar to the incidence rates of female population of Stockholm. In the ERR model with external reference there was no significant difference in the background rates between the exposed and unexposed cohort ($P = 0.36$).

The relatively high excess relative risks observed for the current cohort compared with that in the fluoroscopy studies (Table VI) could perhaps be explained by fractionation effects (Miller *et al.*, 1989; Boice *et al.*, 1991). Such effects were not studied in this report. However, the dose per fraction was comparatively high, probably inhibiting any observable effect of fractionation. In the New York Mastitis Study (Shore *et al.*, 1986) the dose per fraction was also comparatively high and no effect of fractionation was observed. The range of the dose per fraction must perhaps be considerably wider for the effect to be detected in an individual study. In Table VI, estimates at 1 Gy for four different age at first exposure groups from four studies are presented. The study of the A-bomb survivors (Tokunaga *et al.*, 1994) and our study are characterised by one or a few fractions for a given dose. In contrast, the fluoroscopy studies had a given dose delivered in about 100 fractions (Miller *et al.*, 1989; Boice *et al.*, 1991). A marked difference in the ERR possibly depending on the number of fractions is observed. However, this discrepancy could also be due to, for instance, statistical uncertainty, bias or the scale used (additive *vs* multiplicative). If indeed fractionation has an effect, it must be considered in models for the prediction of the excess risk after exposures to low doses distributed in many fractions. Such a fractionation effect is important, for instance in the evaluation of the radiation hazard from mammography screening.

To summarise, the current data set supported a linear dose–response relationship at doses $<5$ Gy. The linear effect was, in the multiplicative model, modified by cell killing and age at first exposure. To reflect the increasing absolute effect by age at risk one extra parameter had to be incorporated into the additive model to achieve the same level of goodness of fit as provided by the multiplicative model.

In the light of these results, and if the dose–response relationship has no threshold, as indicated by current radiobiological knowledge (UNSCEAR, 1993), it seems reasonable to assume that the excess relative risk increases monotonically from very low doses, and probably from the lowest possible dose, to medium dose levels. At high dose levels, cell killing is the dominant factor which gives the dose–response curve a downward slope. The excess breast cancer risk due to radiation exposure to the breast among adult women is sustained, in terms of risk relative to background rates, to at least 80 years of age, and probably lifelong.

**Acknowledgements**

This study was supported by the National Institute of Radiation Protection (Project No. P548/89) and the Cancer Society in Stockholm. We are indebted to Mrs Ulla Cassel for expert assistance in data collection, coding and entering data into the computer; to Professor Timo Hakulinen; Per Hall MD, PhD and Mr Hemming Johansson BA for fruitful discussions and valuable comments on the manuscript; to Mrs Elisabeth Bjurstedt for skilful assistance; and to all helpful people at the different registries.

**References**

BARAL E, LARSSON L-E AND MATTSSON B. (1977). Breast cancer following irradiation of the breast. *Cancer*, **40**, 2905–2910.

BEIR-V. (1990). *Committee on the Biological Effects of Ionizing Radiations. Health Effects of Exposure to Low Levels of Ionizing Radiation. BEIR V.* National Academy Press, Washington, DC.

BOICE Jr JD, PRESTON D, DAVIS FG AND MONSON RR. (1991). Frequent chest X-ray fluoroscopy and breast cancer incidence among tuberculosis patients in Massachusetts. *Radiat. Res.*, **125**, 214–222.

COLEMAN MP, HERMON C AND DOUGLAS A. (1989). *Person-years (PYRS). A Fortran Program for Cohort Study Analysis. IARC Internal Report No. 89/006.* International Agency for Research on Cancer. World Health Organization: Lyon.

HILDRETH NG, SHORE RE AND DVORETSKY PM. (1989). The risk of breast cancer after irradiation of the thymus in infancy. *N. Engl. J. Med.*, **321**, 1281–1284.

ICD-7. (1957). *International Classification of Diseases, Injuries and Causes of Death (ICD-7)*, 1955 revision. World Health Organization: Geneva.

ICRP-23. (1975). *International Commission on Radiological Protection. Reference Man: Anatomical, Physiological and Metabolic Characteristics. ICRP Publication 23.* Pergamon Press: Oxford.

LAND CE, BOICE Jr JD, SHORE RE, NORMAN JE AND TOKUNAGA M. (1980). Breast cancer risk from low-dose exposures to ionizing radiation: results of parallel analysis of three exposed populations of women. *J. Natl Cancer Inst.*, **65**, 353–376.

MATTSSON A, RUDÉN B-I, HALL P, WILKING N AND RUTQVIST LE. (1993). Radiation-induced breast cancer: long-term follow-up of radiation therapy for benign breast disease. *J. Natl Cancer Inst.*, **85**, 1679–1685.

MILLER AB, HOWE GR, SHERMAN GJ, LINDSAY JP, YAFFE MJ, DINNER PJ, RISCH HA AND PRESTON DL. (1989). Mortality from breast cancer after irradiation during fluoroscopic examinations in patients being treated for tuberculosis. *N. Engl. J. Med.*, **321**, 1285–1289.

PRESTON DL, LUBIN JH, PIERCE DA AND McCONNEY ME. (1988–93). *EPICURE. User's guide.* Hirosoft International: Seattle.

SHORE RE, HILDRETH N, WOODARD E, DVORETSKY P, HEMPELMANN L AND PASTERNACK B. (1986). Breast cancer among women given x-ray therapy for acute postpartum mastitis. *J. Natl Cancer Inst.*, **77**, 689–696.

THOMPSON DE, MABUCHI K, RON E, SODA M, TOKUNAGA M, OCHIKUBO S, SUGIMOTO S, IKEDA T, TERASAKI M, IZUMI S AND PRESTON DL. (1994). Cancer incidence in atomic bomb survivors. Part II. Solid tumors, 1958–87. *Radiat. Res.*, **137**, S17–S67.

TOKUNAGA M, LAND CE, TOKUOKA S, NISHIMORI I, SODA M AND AKIBA S. (1994). Incidence of female breast cancer among atomic bomb survivors, 1950–1985. *Radiat. Res.* **138**, 209–223.

UNSCEAR. (1988). *United Nations Scientific Committee on the Effects of Atomic Radiation. Sources, Effects and Risks of Ionizing Radiation. 1988 Report to the General Assembly, with Annexes.* United Nations: New York.

UNSCEAR. (1993). *United Nations Scientific Committee on the Effects of Atomic Radiation. Sources and Effects of Ionizing Radiation. UNSCEAR 1993 Report to the General Assembly, with Scientific Annexes.* United Nations: New York.

### Appendix

### Internal reference models with categorical background variables

In the following table the number of breast cancers in exposed and unexposed subjects, estimates of the linear effect, $\alpha$ in the ERR model $\Sigma\alpha_i D\ \mathrm{Exp}(\beta D)$ and 95% confidence intervals (CI) for $\alpha$ by age at first exposure group ($<30$, 30–39, 40–49, $\geq 50$ years) are presented. Background incidence rates were modelled by attained age (ten categories), calendar period (three categories) and age at diagnosis of benign breast disease (seven categories). The common estimate of $\beta$ was $-0.054$.

| *Age at first exposure (years)* | *No. of breast cancers* Exposed | Unexposed | *Estimate of α* | *95% CI* |
|---|---|---|---|---|
| <30 | 56 | 31 | 2.15 | 1.16–3.85 |
| 30–39 | 61 | 38 | 0.82 | 0.41–1.56 |
| 40–49 | 51 | 19 | 0.61 | 0.25–1.39 |
| ⩾50 | 15 | 7 | 0.37 | 0.05–1.41 |
| All | 183 | 95 | 1.22 | 0.72–1.98 |

For the same specification of the background rates model the categorical quantitative ERR model was estimated to be:

$$\underset{(0.201)}{\mathrm{ERR} = 0.72D}\ \underset{(0.017)}{\mathrm{Exp}[-0.052D]}\ \underset{(0.017)}{\mathrm{Exp}[-0.059(E-40)]}$$

The number within parentheses is the standard error of the estimate.